# Maternal vitamin D in pregnancy and infant's gut microbiota: a systematic review

**DOI:** 10.3389/fped.2023.1248517

**Published:** 2023-10-16

**Authors:** Roghayeh Molani-Gol, Maryam Rafraf

**Affiliations:** ^1^ Student Research Committee, Tabriz University of Medical Sciences, Tabriz, Iran; ^2^ Nutrition Research Center, Department of Community Nutrition, Faculty of Nutrition and Food Science, Tabriz University of Medical Sciences, Tabriz, Iran

**Keywords:** vitamin D, maternal, pregnancy, gut microbiota, infant

## Abstract

**Background:**

An infant's gut microbiome plays a vital role in their health, and various factors can impact their gut microbiota composition. This review aimed to summarize the current knowledge regarding the associations between maternal prenatal supplementation with vitamin D and the composition of infants' gut microbiota.

**Method:**

A comprehensive systematic search was done on Web of Science, Scopus, PubMed, ScienceDirect, and Google Scholar databases without date restrictions until December 2022 using relevant keywords. All relevant original articles in English were eligible for the present review.

**Results:**

Eight articles (two mice, three randomized clinical trials, and three cohort studies) were included in this review. The included mice studies reported that maternal prenatal vitamin D supplementation significantly affects the offspring's gut microbiome composition (such as enhancing the abundance of colonic Bacteroides). Moreover, the included cohort studies revealed a significant association between maternal supplementation with vitamin D during pregnancy and the infant's gut microbiome. However, one-third of clinical trials indicated that vitamin D levels *in utero* could influence the colonization of the microbial community in the infant's gut.

**Conclusion:**

The findings of this review revealed that maternal vitamin D supplementation during pregnancy was linked to an infant's gut microbiome and could impact their gut microbiota composition. However, more studies are warranted to confirm these results.

## Introduction

The human microbiota, especially the gut microbiota, is a diverse and rich community of microbes that plays an essential role in disease and health (1). The gut microbiota has critical roles in early postpartum immune development and in shaping the immune system functions throughout life (2). It has been demonstrated that the changes in microbiota are linked with various immune-mediated diseases (3). Obesity, inflammatory bowel disease, and other metabolic illnesses may result from an imbalance in the composition of the gut microbiome (4, 5).

The homeostasis of intestinal bacteria's important contribution to disease etiology emphasizes the need to recognize modifiable factors influencing the composition of intestinal bacteria (6). Several factors, such as diet, genetics, route of delivery, and antibiotic usage, can affect the diversity of gut microbiota (7, 8). The gut microbiota of an infant is initially seeded by the microbiota of the mother (9). The diet of pregnant and lactating women has been connected with the breastmilk microbiota changes and, as a result, in the gut microbiota of infants, with breastfed infants showing more significant effects than formula-fed infants (10). Recent research indicates that maternal nutrition could influence the shaping of the infant's gut microbiota, lending credence to the potential of maternal nutritional interventions to promote health outcomes and decrease disease risks among infants (11). Dietary nutrient intake, such as vitamin D [1,25(OH)2D], an essential fat-soluble vitamin, is required during pregnancy for the optimal health of the pregnant individual and fetus (12). In addition to the bone health of the offspring, maternal vitamin D levels could impact the other health outcomes of the child (13–15).

According to the current evidence, some of the vitamin D health benefits might be through affecting the gut flora (16–19). As a result, vitamin D may influence the function and structure of the gut microbiome (20, 21). Prenatal vitamin D levels may affect the infant gut microbiome by influencing the maternal microbiome (22). However, a clear overview of the existing evidence for this notion is lacking, and there is uncertainty about whether maternal prenatal supplementation with vitamin D could impact the composition of infants' gut flora. To address this question, a comprehensive systematic search was done to summarize the findings of studies investigating the association between maternal prenatal vitamin D supplementation and infants' gut microbial composition.

## Methods

### Search strategy

This systematic review was performed by considering the Preferred Reporting Items for Systematic Reviews and Meta-Analyses (PRISMA) criteria (23) (Supplementary Table S1). The protocol of the current study was approved and registered by the Research Vice-Chancellor of Tabriz University of Medical Sciences (ethical code: IR.TBZMED.REC.1401.258). Electronic databases of the Web of Sciences, PubMed, ScienceDirect, Scopus, and Google Scholar were searched without date restrictions until December 2022 by the MESH terms and the following keywords: maternal OR pregnancy (mesh) OR “pregnant women” (mesh) OR prenatal OR growth OR mother AND “vitamin D” (mesh) OR Calciferol OR “1,25(OH)2D” OR “1,25-dihydroxyvitamin D” AND “gastrointestinal microbiome” (mesh) OR microbiota (mesh) OR “human microbiome” (mesh) OR microbiome (mesh) OR microflora OR “gut flora” AND infants OR infancy OR neonate OR newborn OR baby OR babies OR offspring. To ensure the inclusion of all eligible studies, a separate search was conducted through Google, and the references of included studies were reviewed. Supplementary Table S2 displays the strategy of search.

### Articles screening and selection criteria

The extracted studies were saved in EndNote software, and duplicate studies were removed. The remaining titles and abstracts were screened to identify studies with the correct scope for the current review. Then, the original full-text English-language articles were selected among the screened articles and critically and separately analyzed for eligibility. All peer-reviewed animal and human studies that addressed the findings regarding the effects or association of maternal prenatal vitamin D supplementation on/with an infant's gut microbiota were eligible for inclusion in this study. Since routine maternal vitamin D supplementation is done during pregnancy and lactation, the cohort studies that evaluated the association between maternal vitamin D levels in pregnancy and the infant's microbiome met the eligibility criteria for inclusion in the present review. The PICOS (Population, Intervention, Comparison, Outcome, and Study Design) principles were used to structure the study's inclusion and exclusion criteria (Table 1), and discrepancies in selecting the included studies were resolved by discussion. PICO criteria for this review were as follows: Population: pregnant individuals and their infants under 2 years old; Intervention: maternal prenatal vitamin D supplementation (different doses); Comparison: placebo or low doses of vitamin D; and Outcome: gut microbiota composition of infants such as Firmicutes, Bacteroidetes, Clostridales, and Lachnobacterium abundance.

**Table 1:** PICOS criteria for inclusion of studies.

Parameter	Inclusion criteria
Participants	Pregnant individuals and their infants under 2 years old
Intervention/correlate	Maternal prenatal vitamin D supplementation (different doses)
Comparison	Placebo or low dose of vitamin D (only applicable for randomized controlled trials)
Outcomes	Gut microbiota composition of infants such as Firmicutes, Bacteroidetes, Actinobacteria, and Proteobacteria abundance.
Study design	Cohort studies Randomized controlled trials Animal studies Published in English, dated up to December 2022

Reviews, abstracts, conference papers, editorials, book chapters, posters, letters, theses, and generic studies were not included. Studies that measured the effects of infants' vitamin D supplementation on their gut microbiota were excluded. Studies that the full texts were not available were also excluded.

### Data extraction

The extracted data from the eligible studies were the following: the authors' name, publication year, study design and location, the number of participants, mean age of infants and mothers, infant sex, maternal supplementation, breastfeeding status, confounders considered in the analysis, and findings concerning the effects of maternal supplementation with vitamin D during pregnancy on infant's gut microbiota (diversity and abundance of bacterial taxa such as Bacteroidetes, Firmicutes, Actinobacteria, and Proteobacteria). Moreover, the method and duration of vitamin D treatment were extracted from the included studies.

### Risk of bias assessment

The quality of the mice studies, cohort studies, and randomized controlled trials (RCTs) was evaluated by the Office of Health Assessment and Translation (OHAT) Handbook (24), the Newcastle–Ottawa scale, and the Cochrane Handbook, respectively. For each risk of bias question, the OHAT tool offers the following response options: definitely a high risk of bias (score = −2), probably a high risk of bias (score = −1), probably a low risk of bias (score =  +1), or definitely a low risk of bias (score =  +2). After answering questions, numerical values were given to each question from −2 to +2, and according to the calculated average for each included study, studies were tiered to three levels of high, moderate, and low risk of bias, using the OHAT recommendations. Based on Approach 1, Tier 1 studies had probably low or low risk of bias, Tier 3 studies had probably high or high risk of bias, and Tier 2 studies had none of Tier 1 or Tier 3 criteria. Approach 2 calculates the overall average ratings of all questions, then using a mean rating per the study, each study is assigned to one of the three tiers based on the cutoffs of more than 0.7, 0.7 to −0.6, and less than −0.6 for Tiers 1–3, respectively (25, 26).

Assessment using the Newcastle–Ottawa scale was divided into three sections: selection, comparability, and outcome or exposure evaluation. Its score for cohort studies had a maximum of 9 points, and the study with an overall score between 6 and 9 points (≥3 points for the selection, 1 point for the comparability, and ≥2 points for the outcome or exposure section) was of good quality (27, 28). The Cochrane Collaboration's tool includes six domains of bias and three risk-of-bias response options: “low,” “high,” and “unclear” (25). Each included study was considered to have a “low risk of bias” if it had a low risk of bias in all three key domains (selection, detection, and performance domains), an “unclear risk of bias” if it had an unclear risk of bias in one or more than one key domain, and a “high risk of bias” if it had a high risk of bias in one or more than one key domain (25, 29).

## Results

### Study selection

**Figure 1:**
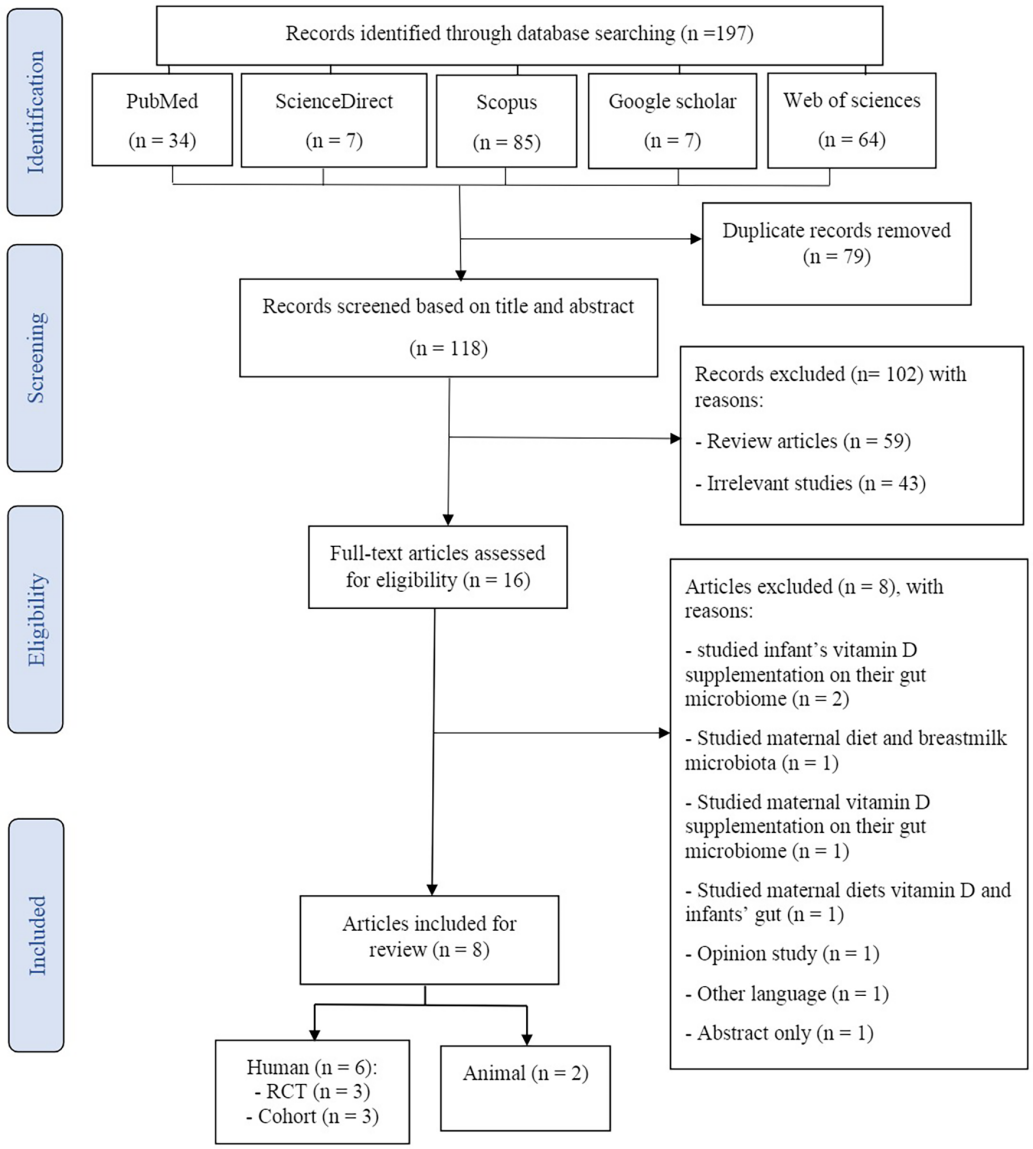
PRISMA diagram for the process of the search and study selection.

The process of search and study selection (PRISMA diagram) for this systematic review is shown in Figure 1. One hundred ninety-seven potential articles were retrieved through searching in PubMed (*n* = 34), Web of Science (*n* = 64), Scopus (*n* = 85), ScienceDirect (*n* = 7), and Google Scholar (*n* = 7) databases. After the elimination of duplicate articles, 118 studies remained for further screening. Of these, 104 studies were excluded based on the title and abstract screening of the articles in the first stage. During critical analysis, 16 articles were screened, of which 8 articles were excluded because these were studying the effects of infants' supplementation with vitamin D on their gut microbiome (*n* = 2) (30, 31), studying the association between maternal diet and breastmilk microbiota (*n* = 1) (32), studying the impacts of maternal vitamin D supplementation on their gut microbiome (*n* = 1) (33), studying the association of maternal diets' vitamin D and infants' gut (*n* = 1) (34), were opinion studies (*n* = 1) (35), were published in other languages (*n* = 1) (36), and were abstract only (*n* = 1) (37). Finally, eight articles (two mice, three RCTs, and two cohorts) were included in this systematic review.

### Characterization of the included studies

Tables 2, 3 present the main characteristics of the included studies. As shown in Table 2, two mice studies (19, 38) included 64 female C57BL/6 J mice that received 5,000 IU (high) and 25 IU (low) oral vitamin D during pregnancy. Three included RCTs were conducted from 2017 to 2020 and involved 1,236 mother–infant pairs. Pregnant individuals in intervention groups received 2,400, 4,000, and 4,400 IU vitamin D daily during week 24 pregnancy to 1 week postpartum (39), second and third trimesters (22), and all pregnancy time (40), respectively, while pregnant individuals in other groups received 400 IU routine vitamin D intake during these times. All women were instructed to continue 400 IU of vitamin D supplementation during pregnancy. In two RCT infants, stool samples were obtained at age 3–6 months (22, 40), and in one case, stool samples were collected on 1 week, 1 month, and 1 year after birth (39). Both sexes had been included in both three RCTs. Exclusively breastfeeding percentage of infants was 17% and 29.41% in two RCTs, and one case has not reported the breastfeeding status of included infants.

**Table 2:** Characteristics of the included animal studies.

First author year (Ref.)	Country	Mice	Age of fecal specimens sampling	*n*/group (maternal-offspring)	VD treatment method	Treatment	Treatment duration	Findings
Li et al. 2023 (19)	China	C57 BL/6 J females and obese male offspring mice	5 months	10/group	Oral	- Control group (VD-C): 5,000 IU VD3/kg diet - VD-deficient group (VD-D): 25 IU VD3/kg diet	Throughout pregnancy and lactation	Maternal deficient VD intake significantly aggravated the dysbiosis of the gut microbiota as follows: - Significantly depleted Bacteroidetes and Verrucomicrobia - Higher relative abundance of Firmicutes and Firmicutes/Bacteroidetes.
Villa et al. 2018 (38)	USA	C57BL/6 J females and male and female offspring	7 months	15/group	Oral	- High vitamin D: 5,000 IU per kg diet - Low vitamin D: 25 IU per kg diet.	From before mating until weaning	Maternal vitamin D effects on colonic Bacteroides but not Prevotella (dam diet: 0.001 and 0.735) in adult male offspring, independent of dams fecal Bacteroides before birth (*p* = 0.998).

VD, vitamin D.

**Table 3:** Characteristics of the included human studies.

Citation (first author et al. year)	Study design/country	Number of participants	Infant sex	Age at stool sample collection	Breastfeeding status	Maternal vitamin D supplementation dose	Maternal vitamin D supplementation duration	Confounders considered in the analysis	Findings of vitamin D supplementation and infants gut microbiome
Hjelmsø et al. 2020 (39)	Randomized factorial clinical trial/Denmark	580 mother–infant	Male/female	1 week, 1 month, and 1 year	Not reported	1) Intervention group: 2,400 IU daily + 400 IU daily 2) Control group: Placebo + 400 IU daily	Pregnancy week 24 to 1 week postpartum	Mode of delivery, older siblings, antibiotics during pregnancy, antibiotics in the first month of life, birth season, cat at home at birth, dog at home at birth, and sex	The vitamin D interventions did not affect the infant's fecal microbiota - Changes in overall beta diversity
Savage et al. 2018 (40)	Randomized clinical trial/USA	323 mother–infant	Male/female	3–6 months	Exclusively breastfed = 95 infants Exclusively formula fed = 169 infants	Intervention: 4,400 IU daily Control: 400 IU daily	During pregnancy	Mode of delivery, child race/ethnicity (African American, Hispanic, white, other), age at stool collection, treatment arm, and maternal education	Maternal diet during pregnancy was less associated with the infant gut microbiome
Sordillo et al. 2017 (22)	Randomized clinical trial/USA	333 mother–infant	Male/female	3–6 months	Exclusively breastfed = 126 (17.0%) infants	1) Intervention: 4,000 IU + prenatal vitamins 2) Control: 400 IU + prenatal vitamins	The second and third trimester of pregnancy	Race, breast-feeding, C-section, sex, maternal antibiotics during labor, gestational age, and treatment group	- Increased abundance of Lachnobacterium - Decreased abundance of Lactococcus - Higher levels of Lachnospiraceae/U. Clostridales in infants with higher cord blood vitamin D
Drall et al. 2020 (41)	Cohort/Canada	1,157 mother-child	Male/Female	3–4 months	Exclusively breastfed = Infant supplement intake ≥400 IU/day: No: 163 (25.19) day Yes: 484 (74.81) day	1) No maternal vitamin D or less than 400 IU/day 2) Prenatal only maternal vitamin D supplementation ≥400 IU/day 3) Postnatal only maternal vitamin D supplementation ≥400 IU/day 4) Prenatal and Postnatal supplementation ≥400 IU/day	During pregnancy and breastfeeding	Maternal milk consumption during pregnancy, household pets, age at stool sample collection, study center, and pre-natal supplement use (maternal and infant)	Among those exclusively breastfed: - Lower abundance of Bilophila and of Lachnospiraceae - Higher abundance of Haemophilus Among partially and not breastfed: - No differences in microbiota composition Maternal consumption of vitamin-D fortified milk reduced the likelihood of *C. difficile* colonization in infants (adjusted OR: 0.40, 95% CI: 0.19–0.82)
Kassem et al. 2020 (42)	Cohort/USA	1,258 mother–child	Not reported	1 and 6 months	Not reported	Not reported	Not reported	Maternal race (Black vs. White, excluding others)	Increasing prenatal 25(OH)D level was significantly associated with decreased richness (*p* = 0.028) and diversity (*p* = 0.012) of the gut microbiota at 1 month of age Both prenatal and cord 25(OH)D were significantly associated with 1 month microbiota composition
Talsness et al. 2017 (43)	Cohort/Netherland	913 mother–child	Male/female	1-month old	Breastfeeding [*n* (%)]: maternal multivitamin supplementation containing vitamin D: None = 243 (69.4) <10 µg = 105 (72.9) ≥10 µg = 268 (64.0)	1) None (0 µg) 2) <400 IU/day 3) ≥400 IU/day	During pregnancy	Place and mode of delivery, number of siblings, recruitment group, total bacterial counts, sex, vaginitis during the last month of pregnancy, and mode of infant nutrition	A statistically significant negative linear trend between counts of Bifidobacterium spp. and levels of maternal vitamin D supplementation and maternal 25-hydroxyvitamin D quintiles A positive linear trend between quintile groups and *B. fragilis* group counts

A total of 3,328 mother–child pairs participated in three included cohort studies. All the cohort studies included both sexes of infants. In included cohort studies, infants' stool samples were obtained at ages 3–4 months (41), 1 and 6 months (42), and 1 month after birth (43). The breastfeeding percentage of infants was 74.81% (41) and 64% (43) in vitamin D-supplemented infants, and one case has not reported the percentage of infants’ breastfeeding. Drall et al. (41) created four categories for maternal supplementation with vitamin D according to the Dietary Reference Intakes and Recommended Dietary Allowances of 400 and 600 IU/day vitamin D supplementation during pregnancy and breastfeeding, respectively (44, 45). Their four maternal supplementations of vitamin D categories are the following: no or less than 400 IU/day supplementation, only prenatal supplementation of ≥400 IU/day, only postnatal supplementation of ≥400 IU/day, and prenatal and postnatal supplementation with ≥400 IU/day vitamin D. In another cohort study also based on maternal multivitamin supplementation containing vitamin D, three categories for maternal vitamin D supplementation were considered, including none supplementation, <400, and ≥400 IU/day (43). All RCTs and cohort studies considered some possible confounders in their analysis.

### Risk of bias assessment

Supplementary Table S3 shows the mouse studies' risk of bias assessment and tier classifications. Based on the OHAT's tier system, two mouse studies were classified as tier 1. One of the mice studies has not reported using of randomization in the mice's allocation into the groups and the blindness of outcome assessors (19), and other mice studies provided unclear data about adequately concealed allocation to study groups (38).

The included RCTs' risk of bias assessment details are described in Supplementary Table S4. Based on the Cochrane collaboration's criteria, all included RCTs had a low risk of bias. The personnel and participants were not blind in one of the RCTs (40), and others have not reported clear data about allocation concealment (39) and blinding of the outcome's assessor (22). The quality assessment of cohort studies resulted in the mean scores of 8 (41, 42) and 9 (43) as good quality (Supplementary Tables S5).

### Outcomes

Table 3 summarizes the findings about vitamin D supplementation effects on offspring's gut flora composition. Moreover, a synoptic view of the main changes in the offspring's gut microbiome after maternal vitamin D administration is presented in Supplementary Table S6. Of eight included studies (two mice, three RCTs, and three cohorts), two mice and four humans (one RCT and three cohorts) studies (75%) demonstrated that maternal vitamin D supplementation during pregnancy was associated with the infant's gut microbiome and could impact gut microbiota composition such as Firmicutes and Bacteroidetes phyla. The description of the included studies is as follows.

Four included studies (one micee, one RCT, and two cohorts) revealed that maternal prenatal supplementation with vitamin D was associated with Firmicutes phylum abundance (19, 22, 41, 42). Li et al. indicated that vitamin D deficiency in the maternal significantly aggravated the dysbiosis of the mice's gut microbiota, with a relatively high abundance of Firmicutes (Lachnoclostridium, Lactobacillus, Ruminiclostridium_9, and Romboutsia), and high Firmicutes/Bacteroidetes ratio (19). Moreover, Sordillo et al. suggested that vitamin D levels *in utero* could impact the colonized microbial community of the infant's gut. They observed that the levels of Lachnospiraceae/U. Clostridales were higher in infants with higher vitamin D levels in cord blood, and multivariate analysis showed an increased Lachnobacterium abundance and a decreased Lactococcus abundance (22). Drall et al. also showed that maternal prenatal supplementation with vitamin D was related to lower Bilophila and Lachnospiraceae abundance but increased Haemophilus abundance in exclusively breastfed offspring (41). Another cohort study reported that prenatal 25(OH)D levels were positively associated with the microbiota composition of 1-month-aged infants including Acinetobacter, Corynebacterium, and Ruminococcus gnavus (42).

Two mouse studies mentioned above also reported that maternal prenatal vitamin D supplementation had an effect on the Bacteroidetes (Bacteroides, Alliprevotella, and Akkermansia) abundant in offspring mice gut microbiome (19, 38). Moreover, Talsness et al., in a cohort study, revealed similar findings. They reported a positive relationship between maternal 25(OH)D quintiles and Bacteroides fragilis group counts in the gut microbiota of 1-month-old infants. In addition, they indicated a negative linear trend between maternal supplementation of vitamin D and maternal 25(OH)D quintiles with Bifidobacterium spp. counts as a genus of Actinobacteria phylum. Their results suggested that vitamin D was associated with several key bacterial taxa abundance within the infants' microbiota (43).

Finally, two RCT studies reported no significant findings (39, 40). Hjelmsø et al. (39) demonstrated that the vitamin D interventions did not affect the infant's fecal microbiota, and Savage et al. (40) indicated a week association between the diet of maternal during pregnancy and the composition of the infant's gut microbiome. Overall, the included RCTs revealed mixed results for the effect of maternal prenatal vitamin D supplementation on the gut microbiome of infants (Table 3).

## Discussion

Gut microbiota has a significant role in health and disease, and it is now recognized as a human organ that influences other organs (46). Several factors, such as dietary nutrients, could change the balance of gut microbiota (47). The present systematic review, using published animal, RCT, and cohort data, provides a comprehensive assessment of the association between maternal supplementation with vitamin D during pregnancy and the infants' gut microbiome. All the included studies, except two cases, demonstrated that maternal vitamin D supplementation during pregnancy was linked with the infants' gut microbiome and could impact the infant's gut microbiota composition.

The typical microbiota involves four main phyla (Actinobacteria, Bacteroidetes, Firmicutes, and Proteobacteria) (48), and the two major bacterial phyla in human feces are Firmicutes and Bacteroidetes (48, 49). Li et al. reported that maternal deficiency in vitamin D intake during pregnancy results in aggravated dysbiosis of the gut microbiota including the depletion of Bacteroidetes and Verrucomicrobia, and enhancement of the Firmicutes abundance and Firmicutes/Bacteroidetes ratio in the offspring's gut microbiota (19). Villa et al. (38) also showed that maternal vitamin D enhanced the abundance of colonic Bacteroides in infants. Thus, maternal vitamin D supplementation could change the infant's microbiota toward an increase in Bacteroidetes and a decrease in Firmicutes. Many Bacteroidota are symbiotic organisms that have adapted well to the digestive tract. Bacteroidota colonizes the infant's gastrointestinal system because nondigestible oligosaccharides in the mother's milk promote the growth of Bacteroides and Bifidobacterium spp. (50). A change in the gut flora composition to higher Bacteroidetes levels and lower Firmicutes levels may benefit the host, whereas an increase in Firmicutes may contribute to gut barrier failure (51, 52).

The association between vitamin D levels and human gut microbiota composition is sufficiently evident (18). The maternal gut microbiota is also linked to maternal dietary vitamin D consumption (33). However, only a few studies have investigated the effects of maternal prenatal vitamin D supplementation on the infants' gut microbiome and the potential links between maternal vitamin D levels and gut flora in early life. Maternal supplementation with vitamin D during pregnancy was negatively associated with Bifidobacterium species counts of 1-month-old infants' gut microbiota in the KOALA birth cohort, while the counts of Clostridioides difficile were reduced in 1-month-old infants' gut flora following prenatal vitamin D supplementation of pregnant individuals (43). Although asymptomatic Clostridioides difficile colonizes approximately 30% of newborns, it has been related to a disruption in gut microbiota composition and later pediatric allergy illness (53). The 25(OH)D levels of cord blood were linked with higher Lachnobacterium levels and lower Lactococcus levels in the Trial of Vitamin D Antenatal Asthma Reduction (22). Drall et al. also showed an association between maternal prenatal and postnatal vitamin D supplementation (≥400 IU/day) and lower Bilophila spp. abundance (41). Bilophila has been associated with colitis and inflammation in mice (54, 55) and colic in infants (56). These results suggest that maternal vitamin D levels could affect several key bacterial taxa in the gut microbiome of infants.

Vitamin D is involved in several physiological processes, including gut microbiota preservation and the exclusion of opportunistic bacteria. The receptors of vitamin D are highly expressed in the intestinal enterocytes, in particular, the proximal colon, and contribute to the production of antimicrobial peptides such as cathelicidins, defensins, claudins, and zonulin occludes (57–59). In other words, vitamin D is a well-known upregulating factor for the gene expression of antimicrobial peptides in various cell types, such as colonic cells (60). Selective killing of pathogenic bacteria would result in greater opportunity for colonization with healthy bacteria. Fermentation products of symbiotic bacteria could upregulate intestinal expression of the vitamin D receptor (VDR) and downregulate inflammation (61). Vitamin D also plays a role in maintaining the mucosal barrier function by upregulating the expression of tight junction and adherent junction proteins and suppressing epithelial cell apoptosis that maintains the integrity and function of the gut barrier (57–59). Furthermore, Li et al. (19) found that proinflammatory cytokine and lipid transport molecule gene expression was greater in the vitamin D deficient group, while intestinal barrier function was lower in their offspring than in the vitamin D control group. There were strong connections between intestinal microbial community dysbiosis and gene expression in the ileum and colon linked with inflammation, barrier function, and lipid transport (19). Because inflammation helps harmful bacteria overcome resident bacteria for colonization resistance, reducing intestinal inflammation promotes microbiota balance (61). Vitamin D is well known to have a pivotal role in intestinal homeostasis by binding with the VDR and subsequently affecting the expressions of relevant genes on the intestinal epithelial barrier and chronic inflammatory state (20). Vitamin D by reinforcing intercellular junctions contributes to maintaining the mucosal barrier function. Disrupting the gut barrier function exacerbates infection with pathogenic bacteria and enhances inflammation, which then has downstream influences on the gut microbiota (18–20). Overall, vitamin D status can alter the gut microbiota because it stimulates anti-inflammatory responses and prevention of infections by the immune system. As a result, vitamin D status is a modifiable factor that is important in the prevention of immune-mediated illnesses by influencing the maintenance of gut microbiota homeostasis (43).

### Strengths and limitations

The inclusion of both animal and human studies in this review makes this study a comprehensive systematic review. However, there were some limitations to this study. The small number of included animal, RCT, and cohort studies was the main limitation of this systematic review. The included RCTs also used different doses of vitamin D. Furthermore, due to the heterogeneity of the results reported among the included studies, it was not possible to perform a meta-analysis. In addition, stool samples were collected at different time points; future studies should collect more samples over the first year of life. In the included studies, infant microbiota was not sampled right after birth. This would have allowed us to detect a possible fecal bacterial transfer event during birth.

## Conclusion

The findings of the present systematic review suggest a significant association between maternal supplementation with vitamin D during pregnancy and the infant's gut microbiota composition. The included animal studies also reported that maternal prenatal vitamin D supplementation can influence the gut microbiota composition of offspring. However, the included clinical trials revealed mixed results. It seems that adequate maternal vitamin D intake during the prenatal period is an important factor in the maintenance of the infant's gut microbiota hemostasis. Further, well-designed RCTs are warranted to confirm the possible effects of maternal prenatal vitamin D supplementation on the infants' gut microbiota. Prospective cohort studies with a large number of infants are needed to examine the association between routine maternal vitamin D treatment during pregnancy and the infant's gut microbiota. Furthermore, mechanistic examinations on the effect of maternal vitamin D levels on the infants' gut microbiome are also suggested.

## Data Availability

The original contributions presented in the study are included in the article/**Supplementary Material**, further inquiries can be directed to the corresponding author.
