# Supplementary material for: Maternal vitamin D in pregnancy and infant's gut microbiota: a systematic review
Source: Front Pediatr. 2023 Oct 16;11:1248517. doi: 10.3389/fped.2023.1248517 (PMC10617198; doi:10.3389/fped.2023.1248517)
Supplement: Supplementary file 2 [file Table2.docx]

**Supplementary Table 2.** Maternal vitamin D in pregnancy and infant’s gut microbiota: A systematic review: Method of the database search strategy using PubMed, Scopus, ScienceDirect, Google Scholar, and Web of Sciences

| **Database (Search**  **conducted up to**  **December, 2022)** | **Search terms^a^** | **Number of studies searched** |
| --- | --- | --- |
| PubMed | (((((((Pregnancy[MeSH Terms]) OR (pregnant women[MeSH Terms])) OR (maternal[Title/Abstract])) OR (prenatal[Title/Abstract])) OR (mother[Title/Abstract])) AND (((((1 alpha, 25 dihydroxy 20 epi vitamin d3[MeSH Terms]) OR (Vitamin D[Title/Abstract])) OR (Calciferol[Title/Abstract])) OR (1,25(OH)2D[Title/Abstract])) OR (1,25-dihydroxyvitamin D[Title/Abstract]))) AND ((((((Microbiota[Title/Abstract]) OR (microbiota[MeSH Terms])) OR (microbiome[MeSH Terms])) OR (microbiome[Title/Abstract])) OR (microflora[Title/Abstract])) OR ("gut flora"[Title/Abstract]))) AND (((((((((infant[Title/Abstract]) OR (neonate[Title/Abstract])) OR (offspring[Title/Abstract])) OR (newborn[Title/Abstract])) OR (baby[Title/Abstract])) OR (babies[Title/Abstract])) OR (infancy[Title/Abstract])) OR (infant[MeSH Terms])) OR (neonate[MeSH Terms])) | 34 |
| SCOPUS | ( ( TITLE-ABS-KEY ( infant ) OR TITLE-ABS-KEY ( infancy ) OR TITLE-ABS-KEY ( neonate ) OR TITLE-ABS-KEY ( newborn ) OR TITLE-ABS-KEY ( offspring ) OR TITLE-ABS-KEY ( baby ) OR TITLE-ABS-KEY ( babies ) ) ) AND ( ( TITLE-ABS-KEY ( microbiome ) OR TITLE-ABS-KEY ( microbiota ) OR TITLE-ABS-KEY ( "gut flora" ) OR TITLE-ABS-KEY ( microflora ) ) ) AND ( ( TITLE-ABS-KEY ( "vitamin D" ) OR TITLE-ABS-KEY ( calciferol ) OR TITLE-ABS-KEY ( 1,25-dihydroxyvitamin AND d ) OR TITLE-ABS-KEY ( "1,25(OH)2D" ) ) ) AND ( ( TITLE-ABS-KEY ( pregnancy ) OR TITLE-ABS-KEY ( maternal ) OR TITLE-ABS-KEY ( mother ) OR TITLE-ABS-KEY ( "pregnant women" ) OR TITLE-ABS-KEY ( prenatal ) ) ) | 85 |
| Google Scholar | allintitle: "vitamin D" AND microbiome AND infant  allintitle: "vitamin D" AND microbiome AND newborn  allintitle: "vitamin D" AND microbiome AND neonate  allintitle: "vitamin D" AND microbiome AND offspring  allintitle: "vitamin D" AND microbiome AND baby  allintitle: "vitamin D" AND microbiome AND infancy  allintitle: "vitamin D" AND microbiota AND infant  allintitle: "vitamin D" AND microbiota AND infancy  allintitle: "vitamin D" AND microbiota AND neonate  allintitle: "vitamin D" AND microbiota AND newborn  allintitle: "vitamin D" AND microbiota AND offspring  allintitle: "vitamin D" AND microbiota AND babies  allintitle: "vitamin D" AND microbiota AND babies  allintitle: "vitamin D" AND microflora AND infant  allintitle: "vitamin D" AND microflora AND infancy  allintitle: "vitamin D" AND microflora AND neonate  allintitle: "vitamin D" AND microflora AND newborn  allintitle: "vitamin D" AND microflora AND offspring  allintitle: "vitamin D" AND microflora AND babies  allintitle: "vitamin D" AND microflora AND babies  allintitle: "vitamin D" AND “gut flora” AND infant  allintitle: "vitamin D" AND “gut flora” AND infancy  allintitle: "vitamin D" AND “gut flora” AND neonate  allintitle: "vitamin D" AND “gut flora” AND newborn  allintitle: "vitamin D" AND “gut flora” AND offspring  allintitle: "vitamin D" AND “gut flora” AND babies  allintitle: "vitamin D" AND “gut flora” AND babies  N= | 2  0  0  0  0  0  1  1  0  0  1  0  0  0  1  0  0  1  0  0  0  0  0  0  0  0  0  7 |
| ScienceDirect | Title, abstract, keywords: "vitamin D" AND ("Microbiota" OR "Microbiome" OR "microflora" OR "gut flora") AND (infant OR neonate OR newborn OR offspring); Article type: research articles | 7 |
| Web of Sciences | #1 TS=(infant OR offspring OR neonate OR newborn OR infancy OR baby OR babies)  #2 TS=(microbiome OR microbiota OR microflora OR "gut flora")  #3 TS=("Vitamin D" OR calciferol OR 1,25(OH)2D OR "1,25-dihydroxyvitamin D")  #4 TS=(pregnancy OR pregnant women OR mother OR maternal OR prenatal)  #1 AND #2 AND #3 AND #4 | 779325  168601  104733  951768  64 |
| Total |  | 197 |

^a^Searches were limited to original articles, and studies published in the English language using the appropriate filters and/or search terms depending on the database.
