# Supplementary material for: Maternal vitamin D in pregnancy and infant's gut microbiota: a systematic review
Source: Front Pediatr. 2023 Oct 16;11:1248517. doi: 10.3389/fped.2023.1248517 (PMC10617198; doi:10.3389/fped.2023.1248517)
Supplement: Supplementary file 3 [file Table3.docx]

| **CATEGORY** | **QUESTIONS** | Li et al. 2023 | Villa et al. 2018 |
| --- | --- | --- | --- |
| Selection Bias | 1. Was administered dose or exposure level adequately randomized? | + | ++ |
|  | 2. Was allocation to study groups adequately concealed? | + | + |
| Performance Bias | 3. Were experimental conditions identical across study groups? | ++ | + |
|  | 4. Were the research personnel and human subjects blinded to the study group during the study? | + | ++ |
| Attrition/Exclusion Bias | 5. Were outcome data complete without attrition or exclusion from analysis? | ++ | ++ |
| Detection Bias | 6. Can we be confident in the exposure characterization? | ++ | ++ |
|  | 7. Can we be confident in the outcome assessment? | ++ | ++ |
| Selective Reporting Bias | 8. Were all measured outcomes reported? | ++ | ++ |
| Other Sources of Bias | 9. Were there no other potential threats to internal validity (e.g., statistical methods were appropriate and researchers adhered to the study protocol)? | ++ | ++ |
| Overall Tier-Approach 1 for tiering | | 1 | 1 |
| 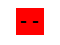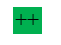Risk of bias response options for individual items:  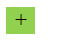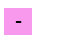 Definitely low risk of bias Definitely high risk of bias Probably low risk of bias Probably high risk of bias | | | |

**Supplementary Table 3:** The risk of bias assessment and tier classifications of animal studies.
