# Supplementary material for: Maternal vitamin D in pregnancy and infant's gut microbiota: a systematic review
Source: Front Pediatr. 2023 Oct 16;11:1248517. doi: 10.3389/fped.2023.1248517 (PMC10617198; doi:10.3389/fped.2023.1248517)
Supplement: Supplementary file 4 [file Table4.docx]

**Supplementary Table 4.** The risk of bias assessment and tier classifications of randomized controlled trials (Cochrane Collaboration's tool)

| **CATEGORY** | **Criteria** | Hjelmsø et al. 2020 | Savage et al. 2018 | Sordillo et al. 2017 |
| --- | --- | --- | --- | --- |
| Selection bias | Random sequence generation | + | + | + |
|  | Allocation concealment | ? | + | + |
| Performance bias | Blinding of participants and personnel | + | ? | + |
| Detection bias | Blinding of outcome assessment | + | + | ? |
| Attrition bias | Incomplete outcome data addressed | + | + | + |
| Reporting bias | Selective reporting | + | + | **+** |
| Other bias | Anything else | + | + | + |
| Overall risk of bias | | + | + | + |
| -  ?  +  Low risk of bias Unclear risk of bias High risk of bias  - | | | | |
