# Supplementary material for: Maternal vitamin D in pregnancy and infant's gut microbiota: a systematic review
Source: Front Pediatr. 2023 Oct 16;11:1248517. doi: 10.3389/fped.2023.1248517 (PMC10617198; doi:10.3389/fped.2023.1248517)
Supplement: Supplementary file 5 [file Table5.docx]

**Supplementary Table 5.** Newcastle-Ottawa scale for assessment of quality of three included cohort studies.

| Study ID | Quality assessment criteria | | | | | | | | | | | | | Overall Quality Score (Maximum = 9) |
| --- | --- | --- | --- | --- | --- | --- | --- | --- | --- | --- | --- | --- | --- | --- |
|  | **Selection** | | | | | | **Comparability** | | **Outcomes** | | | | |  |
|  | Representativeness of exposed cohort? | | Selection of the non-exposed cohort? | Ascertainment of exposure? | | Demonstration that outcome of interest was not present at start of study? | Comparability of cohorts on the basis of the design or analysis controlled for confounders | | Assessment of outcome? | | Was follow-up long enough for outcomes to occur | Adequacy of follow-up of cohorts | |  |
|  | Truly representative | Somewhat representative | Drawn from same community as the exposed cohort | Secured records | Structured interview | yes | The study controls for main confounders | Study controls for other factors | Independent blind assessment | Record linkage | yes | Complete follow up- all subject accounted for | Subjects lost to follow up unlikely to introduce bias- number lost less than or equal to 20% or description of those lost suggested no different from those followed. |  |
| Drall et al. 2020 | * |  | * |  | * | * | * | - | * | - | * | * | NS | 8 |
| Kassem et al. 2020 | * |  | * |  | * | * | * | NS | * | - | * | * | NS | 8 |
| Talsness et al. 2017 | * |  | * |  | * | * | * | * | * | - | * | * | NS | 9 |

*, Acceptable; NS, not stated
