# Supplementary material for: Maternal vitamin D in pregnancy and infant's gut microbiota: a systematic review
Source: Front Pediatr. 2023 Oct 16;11:1248517. doi: 10.3389/fped.2023.1248517 (PMC10617198; doi:10.3389/fped.2023.1248517)
Supplement: Supplementary file 6 [file Table6.docx]

**Supplementary Table 6.** Synoptic view of the main changes of offspring’s gut microbiome after maternal vitamin D administration

| **Hjelmsø 2020 *** | Change beta diversity |  |  |  |  |  |  |  |  |  |  |  |  |
| --- | --- | --- | --- | --- | --- | --- | --- | --- | --- | --- | --- | --- | --- |
| **Savage 2018 *** |  |  |  |  |  |  |  |  |  |  |  |  |  |
| **Sordillo 2017** |  | (+) Lachnobacterium | (-) Lactococcus | (+) Lachnospiraceae | (+) Clostridales |  |  |  |  |  |  |  |  |
| **Drall 2020** |  |  |  | (-) Lachnospiraceae** |  | (-) Bilophila** | (+) Haemophilus** |  |  |  |  |  |  |
| **Kassem 2020** |  |  |  |  |  |  |  | (-) Richness  (-) Diversity |  |  |  |  |  |
| **Talsness 2017** |  |  |  |  |  |  |  |  | (-) Bifidobacterium | (+)  B.fragilis |  |  |  |
| **Li 2023***** |  |  |  |  |  |  |  |  |  |  | (+) Bacteroidetes | (-) Firmicutes | (+) Verrucomicrobia |
| **Villa 2018***** |  |  |  |  |  |  |  |  |  |  | (+) Bacteroides |  |  |

* Vtamin D interventions did not affect the infant's fecal microbiota. ** In breastfed. *** Animal study

(+) increased (-) decreased
